# Dental Health Benefits of Swimming in Chlorinated Water

**DOI:** 10.3390/dj12040087

**Published:** 2024-03-31

**Authors:** Barbara Sophie Gaugeler, Jan Gerrit van der Stouwe, Christian Templin, Christian M. Schmied, Martin Lanzer, David Niederseer

**Affiliations:** 1 Ordination Dr. Lanzer, St. Peter Hauptstraße 27, 8042 Graz, Austria; barbara@zahn-gaugeler.at; 2 Cardiology, University Hospital Basel, 4031 Basel, Switzerland; 3 Department of Cardiology, University Heart Center, University Hospital Zurich, University of Zurich, 8006 Zurich, Switzerland; 4 Clinic of Maxillofacial and Oral Surgery, University Hospital Zurich, 8091 Zurich, Switzerland; dr.lanzer@sailerclinic.com; 5 Hochgebirgsklinik, Medicine Campus Davos, 7265 Davos, Switzerland; 6 Christine Kühne Center for Allergy Research and Education (CK-CARE), Medicine Campus Davos, 7265 Davos, Switzerland

**Keywords:** tooth decay, oral health, caries, chlorinated water, swimming, competitive athletes

## Abstract

Poor oral health is an important concern for athletes, as it can affect both general health and athletic performance. The aim of this study is to investigate the effects of activity in chlorinated water on oral health in elite swimmers compared to non-swimming athletes. This cross-sectional study included 101 swimmers and 100 other athletes aged 13–26 years with a minimum training intensity of five hours per week (for at least the preceding two years). Oral health was assessed using the approximal plaque index (API) and the decayed/missing/filled teeth (DMFT) index. A DIAGNOcam was used to detect caries. Results show that swimmers were younger (15 years vs. 18 years), were more likely to be female (54% vs. 17%), and had a lower body mass index (20.1 kg/m^2^ vs. 21.9 kg/m^2^) and a lower juice consumption (9% vs. 24%). Non-swimmers had significantly more decayed, missing, or filled teeth due to caries and plaque. In conclusion, by comparing elite swimmers and athletes competing in different sports, we have shown that competitive swimmers have a lower incidence of dental caries and plaque. Further research is needed to test our findings and to understand this relationship in greater detail.

## 1. Introduction

Oral health, defined as the full functionality and freedom from inflammation or discomfort of all organs in the oral cavity, is an essential determinant of general health [1]. A number of factors influence oral health. These are all well established in the literature, ranging from environmental factors, such as modern-day high-sugar diets, oral hygiene, stress, smoking, and medication, to internal factors, such as the diversity of the oral microbiome, maternal transmission, genetics, and the influence of other systemic diseases [2].

Poor oral health is not an isolated health issue. Instead, it affects general health in many areas: When oral hygiene is compromised, the bacteria in the dental plaque trigger an inflammatory process that transforms the marginal gingiva into a periodontal pocket [3]. This inflammation increases the body’s constitutive inflammatory burden, which influences chronic degenerative diseases such as diabetes mellitus and arteriosclerosis [4]. The influence of the oral microbiota extends far beyond the oral cavity, having effects on the placenta, brain, lungs, heart, and gut [2]. Poor oral health can weaken the body’s immune response in these organs and adversely affect the course of any disease that may occur [2].

Dental caries is one of the most common chronic diseases in children worldwide—even in highly developed countries [5]. For example, Germany is one of the world’s leading countries in terms of dental standards, but up to 20% of children under the age of 12 suffer from dental caries [6] despite the fact that dental caries can be managed by good brushing habits, the use of fluoride toothpaste, and the control of sugar intake, both in terms of frequency and amount [7]. Regular mouth rinsing can also contribute to better oral hygiene simply by diluting oral bacteria. Despite these simple measures, tooth decay affects young children and may persist throughout their lives, continuing into adolescence, adulthood, and even later stages of life [8]. It causes pain and has a negative impact on eating ability, social functioning, and quality of life [9]. For individuals as well as society as a whole, caries also represents a significant financial burden [8]. This burden may well be lessened by a number of simple preventative measures: the promotion of early dental education and regular check-ups could play a crucial role in the prevention and management of dental caries, potentially reducing its prevalence and societal impact in the long term.

Swimming, on the other hand, has many health benefits. The activity can improve cardiovascular fitness [10] and lung function [11], while it has little or no impact on the joints. However, studies have shown that athletes are generally prone to poor oral hygiene [12,13] due to frequent consumption of snacks and drinks as well as low saliva production [14,15]. However, it is currently unclear if this also applies to elite swimmers, who spend a large amount of time in chlorinated water. Hence, the question arises as to whether their activity in the water provides benefits with respect to oral health.

We hypothesise that exposure to chlorine, continuous rinsing of the mouth during training, and dilution of oral bacteria have a beneficial effect on the dental health of swimmers. The objective of this study is to examine the impact of activity in chlorinated water on oral health in elite swimmers in comparison to non-swimming athletes. In particular, our findings show that activity in chlorinated water influences the dynamic process of demineralisation and remineralisation of tooth structure, leading to reduced formation of new plaque, less caries, and an overall improved oral health in elite swimmers.

Additionally, considering the potential implications of oral health on athletic performance and overall well-being, it is imperative for coaches and officials in elite sports to prioritise the promotion of oral health and provide necessary support to athletes. By integrating comprehensive oral health education and preventive measures into athlete support programmes, the athletic community should strive towards fostering not only physical excellence but also holistic well-being among its members. As oral health improves not only general health but also performance, this could serve as a compelling incentive for athletes to prioritise their oral health and adopt healthier lifestyles.

## 2. Materials and Methods

In this cross-sectional study, we compared a group of elite swimmers with non-swimmers of the same age who train at the same intensity to assess the effect of activity in chlorinated water on oral hygiene. This study was conducted between 2020 and 2023 after receiving approval from the Ethics Committee of the Medical University of Graz. Study participants were at least 13 years old and had to have completed the transition to adult dentition. To exclude age-related deterioration in oral health, only athletes up to the age of 26 were included in the study. One hundred consecutive athletes were randomly selected for inclusion in the two groups: The first group consisted of competitive swimmers. The comparison group (non-swimmers) consisted of athletes competing in different sports. Assuming a 10% difference in dental health between the two groups (gamma according to the literature), the required number of cases was calculated based on alpha = 0.05 and beta = 0.95, resulting in 4800 examination units. This corresponds to a subject number of 200, based on 24 teeth in the dentition.

All study participants had to have trained for at least five hours per week over a two-year period (in the case of swimmers, in water). Training history was assessed by interview. Exclusion criteria included training for fewer than five hours per week, training for fewer than two years, participants younger than 13 years or older than 26 years, increased caries risk due to underlying diseases, and lack of parental consent (in case of underage participants) to participate in the study. All participants grew up in Austria, Germany, or Switzerland.

**Figure 1:**
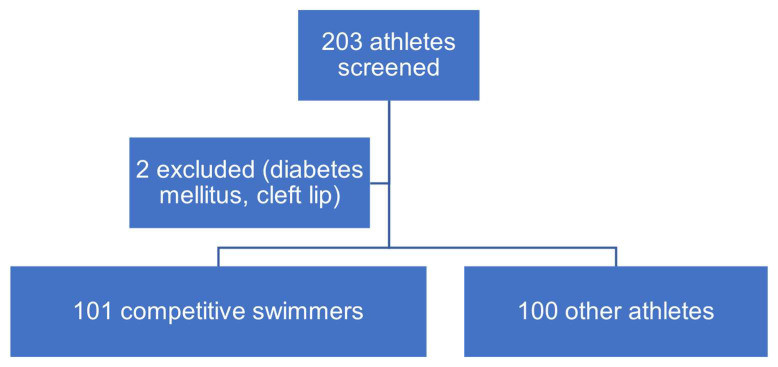
Flowchart of included athletes.

After finishing dental school, B. Gaugeler examined all participants. A total of 102 competitive swimmers and 101 non-swimmers were interviewed and examined. Two participants were excluded because of conditions that increase the likelihood of caries (diabetes mellitus and cleft lip). Therefore, 101 swimmers and 100 non-swimmers were included in the analysis (Figure 1).

At the beginning of our study, the subjects were informed about the study’s procedures. In addition to general and specific medical history, information was gathered on training intensity and history, eating, snacking and drinking habits, as well as oral hygiene. In addition, participants were asked about their occupation and the educational level of their parents because of the relationship between dental health and social status.

The following parameters were collected: the approximal plaque index and the DMFT index, which is a composite of caries (D), missing teeth due to caries (M), and filled teeth (F), indicating a history of caries [6]. 

Plaque was detected using Miradent Plaquetest tablets. Plaque staining was used for the approximal plaque index according to Lange (API) [16] (p. 68). The index scoring procedure was assessed by a yes/no decision per tooth, with the first and third quadrants scored from the oral side and the second and fourth quadrants scored from the buccal side. The result of the API (in %) was calculated using the formula below:

(sum of the positive plaque measurements) × 100 ÷ (number of measuring points)

The scoring is classified as follows:

<5%—optimal oral hygiene;
5–35%—good oral hygiene, improvement possible;
35–50%—oral hygiene needs improvement;
50–70%—poor oral hygiene;
>70%—inadequate oral hygiene.

The DMFT index describes teeth (T = teeth) that have a carious lesion (D = decayed), that have been extracted due to caries (M = missing), or that have been restored with a filling after restorative therapy of a carious lesion (F = filled) [17]. As wisdom teeth are not included, the maximum value in the permanent dentition is 28. Because of its worldwide application, the DMFT index allows for the comparison of epidemiological studies. The D in DMFT stands for dentin lesion. The earlier stages of decay are not taken into consideration [18].

**Figure 2:**
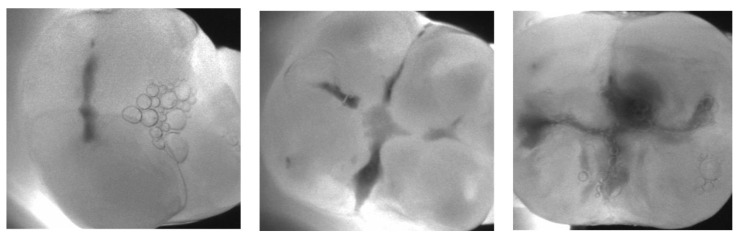
Pictorial representation of increasing caries levels with the help of the DIAGNOcam.

The DMFT index was determined using a KaVo DIAGNOcam 2170 in addition to a clinical dental examination to detect caries. This non-invasive medical device provides an accurate caries diagnosis without the need for X-rays, using DIFOTI (Digital Imaging Fibreoptic Transillumination) technology. The tooth structure allows light to pass from the point of entry to the camera. Areas that block the transmission of light (e.g., carious lesions) are clearly shown in a darker colour (Figure 2). Providing accurate caries detection, the DIAGNOcam eliminated the need for participants to visit a dental clinic.

Finally, we checked for the presence of tooth abrasions, mucosal changes, increased chewing muscle tone (which would have indicated bad oral hygiene habits), and temporomandibular joint symptoms. Participants were asked if they had suffered dental trauma during sports.

Baseline characteristics included the distinction between swimmers and non-swimmers. After checking distribution, continuous variables were presented as median (interquartile range) or mean (±standard deviation) and were compared using the Kruskal–Wallis test or analysis of variance, respectively. The chi-squared test was used to compare categorical variables.

As the count data showed overdispersion, negative binomial regression was used instead of Poisson regression to model the association of swimming with the DMFT index and API. The analyses were adjusted for a pre-selected set of covariates including sex, age, hours of training per week, parental education level, daily flossing, and dietary habits (number of sweets per day, number of meals per day, and regular juice consumption). The parents’ education was entered as a categorical variable according to higher education level. Sweets consumption, daily meals, and regular juice consumption were also entered as categorical variables. These were defined as no sweets or one or more sweets daily, more than three meals daily, and preference for juice over water as the main drink, respectively.

SPSS version 26 (IBM Corp. Armonk, New York, NY, USA) was used for all statistical analyses. A *p*-value of <0.05 was pre-defined to indicate statistical significance.

## 3. Results

A total of 201 participants (101 swimmers and 100 non-swimmers) were included in this study. Baseline characteristics are presented in Table 1. The swimmers analysed in this study are younger (15 years vs. 18 years, *p* < 0.001) and more likely to be female (54% vs. 17%, *p* < 0.001). Body mass index is lower in swimmers compared to non-swimmers (20.1 kg/m^2^ vs. 21.9 kg/m^2^, *p* = 0.004). Weekly training hours and parental education do not differ between the groups. The only statistically significant difference in dietary habits is daily juice consumption, which is higher in non-swimmers (9% vs. 24%, *p* = 0.004).

**Figure 3:**
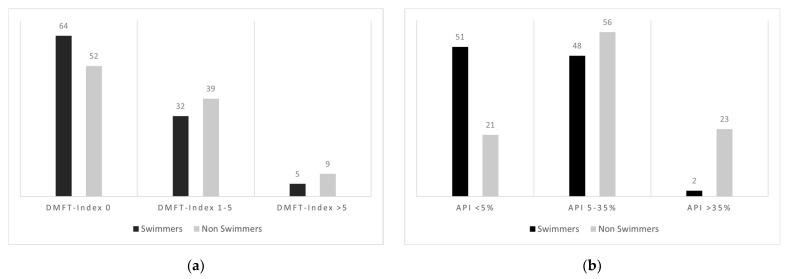
(**a**) Distribution of DMFT index between swimmers and non-swimmers. Abbreviations: DMFT = decayed/missing/filled teeth. (**b**) Distribution of API between swimmers and non-swimmers. Abbreviations: API = approximal plaque index.

Dental assessment shows that only 37% of swimmers have a DMFT index ≥ 1, whereas 48% of non-swimmers have a DMFT ≥ 1 (Figure 3a).

A DMFT >5 is almost twice as common in non-swimmers as in swimmers (9% vs. 5%). An API <5% is much more common in swimmers compared to non-swimmers (50% vs. 21%), while only 2% of swimmers have an API >35% compared to 23% of non-swimmers (Figure 3b).

Regression analyses between swimming and the DMFT index are shown in Table 2a.

Non-swimmers are significantly more likely to have decayed, missing, or filled teeth due to caries as assessed by the DMFT index compared to swimmers in a univariable model (exponential β coefficient: 1.60 (95% CI: 1.11–2.31)). In a predefined multivariable model adjusting for sex (categorical), age (continuous), training hours per week (continuous), parental education level (categorical), meals per day (categorical), main type of drink consumed (categorical), sweets (categorical), and flossing (categorical), this association remains significant with an exponential β coefficient of 1.67 (95% CI: 1.06–2.65). Similar results have been obtained for API, as shown in Table 2b.

Non-swimmers are significantly more likely to have plaque than swimmers in a univariable model (exponential β coefficient: 2.91 (95% CI: 1.69–2.71)). This association remains significant in the multivariable model (exponential beta coefficient: 2.47 (95% CI: 2.08–2.94)).

## 4. Discussion

In this representative cross-sectional study, comparing high-performance athletes who compete in different sports, we show that competitive swimmers, who spend an average time of 12 h training in chlorinated water, have a lower incidence of tooth decay and plaque than athletes in other competitive sports. These results remain stable when considering differences in age, sex, and eating habits.

Oral health in the context of competitive sports is described as inadequate in a number of previous studies [12,13,19,20,21]. This has been linked to a lack of knowledge as well as neglect of the importance of oral hygiene. Additional contributing factors include high and frequent consumption of drinks which are rich in carbohydrates, acid production, and clenching or pressing during sporting activities. Poor oral health can have a negative impact on elite athletic performance [22]. Firstly, an acute infection can have an immediate impact on an athlete’s training schedule which includes any competitions in which he or she is expected to participate. Secondly, chronic inflammation can affect their recovery, nutritional intake, metabolism, and physical and mental performance. Subacute bacterial infections, which arise from the oral microbiome in dental caries, are likely to constitute a neglected cause of inflammation. Oral pathogens are influenced by intrinsic factors such as exercise-induced immune suppression and reduced salivary flow due to intense training and stress [14,15,23]. However, the effect of these pathogens on the immune system remains unknown. Recognising the multifaceted impact of poor oral health on athletes highlights the importance of implementing comprehensive oral health management strategies that are tailored to the unique needs and challenges of the community of athletes.

Oral health is essential for the whole organism. The mouth is a gateway for bacteria, which has a substantial impact on systemic health beyond the oral cavity. One of the most frequently diseases encountered in the oral cavity is caries, an infectious disease caused by a disturbance in the normal balance of demineralisation and remineralisation between the tooth surface, saliva, and pathogenic bacteria [24] (pp. 112, 113). The process of tooth decay is dynamic. The periods of demineralisation and remineralisation are related to changes in the pH value of the dental biofilm [8]. Some oral pathogenic microorganisms in dental plaque produce acids by fermenting simple carbohydrates [25]. Constant sugar intake, therefore, leads to acidic conditions that have to be neutralised first by the saliva [5]. Continued high sugar intake and reduced salivary flow or poor oral hygiene leads to increased demineralisation. Subsequently, tooth decay can occur [6,26]. However, several hundreds of microorganisms that are beneficial to the body have also been found in the oral cavity [27]. Therefore, regular tooth brushing is not merely important for the removal of plaque and the provision of active ingredients such as fluoride. It has a significant impact on the functional state of the microbiome in the mouth and digestive tract [4]. Given that caries can remain asymptomatic for extended periods until a cavity develops, early prevention and detection through non-invasive techniques are paramount in averting significant dental damage and alleviating associated pain [28]. Emphasising the importance of proactive oral health practices and regular dental check-ups can reduce the risk of developing dental caries and improve overall oral well-being.

The lifestyle of a professional athlete carries with it some risk factors for the development of dental disease. These include additional meals to maintain energy levels and the consumption of high-sugar snacks and acidic sports drinks, which often compromise the athlete’s oral health [22]. The use of these supplements is common because a number of brands targeted both at elite and amateur athletes promise an improvement in performance if their products are consumed regularly during as well as before and after training. But many athletes are not well informed about the consequences for their oral health [29]. Therefore, it is crucial for coaches and sporting associations to invest in enhanced education. Additionally, awareness campaigns should be aimed at athletes to tackle these knowledge gaps and encourage healthier eating habits that promote optimal oral health.

In our study, the demographics do not suggest a difference in knowledge about oral health between the two groups, as their lifestyles, including brushing habits, do not differ. A possible explanation for our findings could simply be that swimmers rinse their mouths more often because they spend a significant period of time in the water. However, rinsing the mouth with water alone does not contribute to plaque reduction [30]. Previous studies have shown that water does not remove plaque from dentures, but chlorine solutions used to disinfect swimming pools do [31]. Importantly, the environment in the mouth is different from that of dentures, as some bacteria (e.g., streptococci, actinomycetes) use adhesins, i.e., special surface molecules, to attach themselves to receptors on the surface of tooth enamel [16] (p. 24). This is the reason why mouthwashes containing chlorhexidine prove to be most successful at removing plaque [32].

Previous studies have investigated the adverse effects of swimming in chlorinated water, including an increased risk of erosion [33,34], calculus, and dental staining [35,36], which are exacerbated by poor pool maintenance [37]. However, the positive effect of chlorinated water on cariogenicity has not yet been confirmed. While existing plaque cannot be removed by rinsing alone, we are the first to show that the formation of new plaque is reduced in swimmers who spend several hours per week training in chlorinated water. It is this prevention of the formation of new plaque (as well as the reduced cariogenicity, as indicated by our study, in the swimmers studied) that contributes to an overall improvement of oral health. Additionally, investigating the mechanisms that contribute to the decrease in plaque formation, such as the interaction between chlorine and oral microbiota, could offer valuable insights into new approaches for preventing dental caries in athletes who are exposed to chlorinated water environments.

There is one previous study that has compared the oral health of competitive and non-competitive swimmers in a similar manner [9]. Seemingly at odds with the results of our study, its clinical examination indices did not show any significant differences between the two groups. However, the study has one crucial limitation: all participants were swimmers with the only difference consisting of the intensity of their training (2.02 ± 0.09 h, 5 times per week vs. 2.03 ± 0.18 h per week). Without a comparison group consisting of non-swimmers, their lack of findings on the effect of chlorinated water on cariogenicity appears less significant and has to be considered in relation to the study’s primary objective: the microbiological and immunological analysis of saliva samples. Having collected saliva samples before and after training, the study found a significant decrease in both saliva IgA and cariogenic microorganisms. This decrease in cariogenic microorganisms is, again, in line with our findings of reduced cariogenicity.

The strength of our cross-sectional study lies in its methods: 201 consecutive participants were examined by one person, reducing interobserver variability. In addition, the latest DIAGNOcam technology was used to provide an accurate objective image of the tooth structure, as shown above in Figure 2.

Several limitations should be noted. Due to the nature of this study, baseline characteristics were not balanced, and residual confounding cannot be excluded or corrected in regression models. Individuals in the swimmer group are, on average, younger and more likely to be female. Data suggest that men tend to place less emphasis on their oral hygiene habits and dental prophylaxis than women [38]. In addition, the participants in our comparison group reported drinking significantly more juice than the swimmers studied, which significantly increases the risk of tooth decay [39]. However, when these noted differences were corrected in a multivariable model, the association between swimming and the DMFT index remained consistent.

In DMFT, we consider each tooth as a single unit (T). An adult can have a maximum value of 28, as the wisdom teeth are not included. On the other hand, DMFS considers each surface (S), making it more informative about the progression of caries, with a maximum value of 128. However, according to the Fifth German Oral Health Study, DMFT is a good measure of caries prevalence and we have chosen to use DMFT as it is easy to apply and has a high level of reproducibility [6].

A further limitation is the lack of investigation of the enamel demineralisation process. In our study, this could not be performed, since blow drying of participants’ tooth surfaces was not possible, considering the assessment of the participants at their competition venues. It should be noted, however, that having one dentist travel to a number of competition venues, rather than asking over 200 participants to book individual appointments at one dental practice, proved to be highly efficacious and allowed for us to collect data within a relatively small time window. The association of change in saliva fluid, saliva IgA factors, high sugar intake, and acid environment in high-performing athletes is reasonable but has not been published yet.

In conclusion, in this representative cross-sectional study, comparing elite swimmers and athletes competing in different sports, we have shown that competitive swimmers have a lower incidence of dental caries and plaque compared to high-performance athletes participating in other sports. These findings underscore the potential beneficial impact of chlorinated water on dental health among elite athletes, suggesting a need for further exploration of its mechanisms and implications. It would be interesting to investigate the effect of chlorinated water on the oral microbiome, the balance of which plays an important role in athletes’ overall health. The change in saliva quantity and composition, which is essential for maintaining oral health, should also be investigated in swimmers.

Dental education is needed to increase awareness of the risk of caries and erosive damage [13] due to constant sugar and acid intake during training. Additionally, the benefits of fluoridated toothpaste and proper prophylaxis should be promoted [40,41]. This is of particular interest to all athletes as dental health not only affects general health but also athletic performance [22].

## Figures and Tables

**Table 1:** Baseline characteristics of swimmers and non-swimmers.

	Swimmers n = 101	Non-Swimmers n = 100	*p* Value
Patient characteristics *			
Age (years)	15 (14–17)	18 (16–21)	<0.001
% Female	54 (54%)	17 (17%)	<0.001
BMI (kg/m^2^)	20.1 (19.1–22.1)	21.9 (19.7–23.4)	0.004
Training per Week (hours)	12 (8–14)	9 (7–14)	0.11
Higher education of parent	44 (44%)	45 (45%)	0.84
Eating habits			
Sweets (never)	47 (47%)	55 (55%)	0.38
Sweets (once daily)	40 (40%)	36 (36%)	
Sweets (multiple daily)	14 (14%)	9 (9%)	
>3 daily meals	47 (47%)	44 (44%)	0.72
Daily juice intake	9 (9%)	24 (24%)	0.004
Oral hygiene habits			
Daily flossing	55 (55%)	45 (45%)	0.18
Teeth brushing (weekly)	14 (14–14)	14 (14–14)	0.05

* Data are shown as mean and standard deviation or median and interquartile range if skewed. Categorical data are shown as numbers and percentages. *p*-values are based on Kruskal–Wallis, ANOVA, or chi-squared tests, as appropriate. Abbreviations: BMI = body mass index; DMFT = decayed/missing/filled teeth.

**Table 2:** (**a**). Regression analyses between swimmers and non-swimmers for DMFT. (**b**). Regression analyses between swimmers and non-swimmers for API.

(a)		
	exp. Β (95% CI)	*p* Value
a Univariable Model	1.60 (1.11–2.31)	0.01
b Multivariable Model	1.67 (1.06–2.65)	0.03 ^1^
**(b)**		
	**exp. Β (95% CI)**	***p * Value **
a Univariable Model	2.91 (1.69–2.71)	<0.001
b Multivariable Model	2.47 (2.08–2.94)	<0.001

^1^ Hazard ratios along with 95% confidence intervals for the association of swimming and the DMFT index. Hazard ratios along with 95% confidence intervals for the association of swimming and API. Abbreviations: DMFT = decayed/missing/filled teeth; API = approximal plaque index; exp. β = exponential beta coefficient. a = univariable regression model; b = includes sex (categorical) and age (continuous), training per week in hours (continuous), parental education level (categorical), meals per day (categorical), main type of beverage consumed (categorical), sweets (categorical), and flossing (categorical).

## Data Availability

The raw data supporting the conclusions of this article will be made available by the authors on request.
